# Impact of the FIGO 2023 Staging System on the Adjuvant Treatment of Endometrial Cancer: A Comparative Analysis with FIGO 2009

**DOI:** 10.3390/cancers17060934

**Published:** 2025-03-10

**Authors:** Federico Ferrari, Elisa Gozzini, Jacopo Conforti, Andrea Giannini, Fabio Barra, Anna Fichera, Filippo Alberto Ferrari, Hooman Soleymani majd, Franco Odicino

**Affiliations:** 1 Department of Clinical and Experimental Sciences, University of Brescia, 25136 Brescia, Italy; 2 Unit of Gynecology, Sant’Andrea Hospital, Department of Surgical and Medical Sciences and Translational Medicine, Sapienza University of Rome, 00189 Rome, Italy; andrea.giannini@uniroma1.it; 3 Unit of Obstetrics and Gynecology, P.O. “Ospedale del Tigullio”—ASL4, Via Gio Batta Ghio 9, 16043 Genoa, Italy; 4 Department of Obstetrics and Gynecology, Gynecologic Oncology and Minimally Invasive Pelvic Surgery, International School of Surgical Anatomy (ISSA), IRCCS “Sacro Cuore—Don Calabria” Hospital, 37024 Verona, Italy; 5 Department of Gynaecology Oncology, Oxford University Hospitals NHS Trust, Oxford OX3 7LE, UK; hooman.soleymani@ouh.nhs.uk

**Keywords:** ProMisE, endometrial cancer, FIGO staging system, adjuvant treatment

## Abstract

**Background:** ProMisE (Proactive Molecular Risk Classifier for Endometrial Cancer) has brought about the publication of the FIGO 2023 staging system with a significant impact in practice for endometrial cancer (EC). **Methods:** In this comparative study, we investigated the need for adjuvant treatment according to FIGO 2023, for each FIGO 2009 stage. We prospectively enrolled EC patients from June 2021 to June 2024, collected classical risk factors and classified according to FIGO 2009. Afterwards, we reclassified the same women according to FIGO 2023 and grouped them according to ESMO/ESTRO/ESGO risk factors. **Results:** We collected 211 eligible patients. We found 176 patients with FIGO 2009 I–II and 35 women with FIGO 2009 III–IV. Based on classic anatomopathological risk factors and FIGO 2009, adjuvant treatment was indicated in 124 (70.5%) patients with FIGO 2009 I–II (vaginal brachytherapy = 48; external beam radiotherapy with or without vaginal brachytherapy = 63; chemotherapy with external beam radiotherapy with or without vaginal brachytherapy = 13) and in 35 (100%) women with FIGO 2009 III–IV (all underwent chemotherapy). After FIGO 2023 re-staging, the women with early-stage EC, were more frequently candidates for no treatment, with a decreased relative risk (RR) for adjuvant treatment (RR 0.84; CI95% 0.74–0.95; *p* < 0.01) when compared to the previous FIGO classification. No significant risk (RR 1.01; CI95% 0.76–1.3; *p* = 0.08) for a more aggressive adjuvant treatment was noted after FIGO 2023 re-staging. In FIGO stage III–IV, no changes in the treatment modalities were observed. **Conclusions:** The re-staging according to FIGO 2023 of women previously staged to FIGO 2009 I–II, yields an RR of 0.84 to spare any type of adjuvant treatment.

## Simple Summary

The FIGO 2023 staging of endometrial cancer definitively introduced molecular classification into the staging system of endometrial cancer pathology. In our work, we evaluated the impact of the new staging on the type of adjuvant treatment in a group of 211 women undergoing surgical treatment for endometrial neoplasia and reclassified according to FIGO 2023 staging. After restaging, the risk of receiving adjuvant treatment is reduced by 16% for early-stage patients, while for advanced stages there is no substantial difference in the need for adjuvant therapy compared with the previous staging.

## 1. Introduction

Endometrial cancer (EC) is the most common gynecologic malignancy in high-income countries [1] and the incidence is expected to increase due to the risk factors associated with the disease [2]. For decades, endometrial cancers have been classified into type I or type II based on Bokhman’s classification [3]. In 2013, The Cancer Genome Atlas (TCGA) Research Network analyzed the genomic profiles of 373 endometrial carcinomas. Based on various characteristics, such as somatic nucleotide substitutions, microsatellite instability (MSI), and somatic copy number alterations (SCNAs), four distinct groups were identified: the ultra-mutated group, characterized by the presence of POLE gene mutations; the hypermutated group, associated with mismatch repair deficiency; the “copy number high” group, defined by p53 gene mutations; and the “copy number low” group, lacking specific molecular features [4]. This analysis allows us to break down EC into four classes: POL-E mutated (POLE-mut); 8–10% of tumors, mismatch repair deficient (MMR-d); 25–30% of neoplasms, P53-abnormal (p53-abn)—copy number high (9% of tumors) and P53-wild type, copy number low, which is the largest group (NSMP) [5].

Two years later, the Proactive Molecular Risk Classifier for Endometrial Cancer (ProMisE) group proposed to adopt the TCGA molecular classification for clinical practice. They achieved this by employing immunohistochemical analysis of p53 and mismatch repair proteins as proxies for detecting TP53 gene mutations and microsatellite instability, respectively. However, for POLE mutations, sequencing of the exonuclease domain remains the only method to accurately identify pathogenic variants, as no alternative surrogate exists. The discovery of these molecular characteristics has profoundly influenced the understanding of the pathology, as the molecular class represents an independent risk factor in terms of disease-free survival (PFS), overall survival (OS) [6] and the probability of recurrence [7,8]. For this reason, the 2020 European Society of Gynaecological Oncology (ESGO), the European SocieTy for Radiotherapy and Oncology (ESTRO), and the European Society of Pathology (ESP) guidelines have updated recommendations for adjuvant therapy; in particular, the positive prognostic role of POL-E and the negative prognostic impact of the p53 mutation are highlighted, considering any post-surgical adjuvant therapy [9,10].

In light of these new findings, in 2023, the International Federation of Gynecology and Obstetrics (FIGO) published the new EC staging system, in which the division is not only based on the anatomical extension of the disease but also on the presence of the POL-E and P53 mutation, of substantial lymphovascular space invasion (LVSI) or of histotype (endometrioid G1/2 vs. endometrioid G3 and other aggressive histotypes) [11]. The aim of our study is to evaluate the impact of the new staging system on clinical practice in early and advanced endometrial cancer, previously classified according to the FIGO 2009 system, with particular focus on adjuvant therapy.

## 2. Material and Methods

We enrolled patients treated for endometrial cancer (EC) between June 2021 and June 2024 at our referral center for gynecologic malignancies. All the data used in this manuscript derive from auditing databases in service evaluation for endometrial cancer and is hence already anonymized at the moment of data extraction. The design, analysis, interpretation of data, drafting, and revisions conform to the Helsinki Declaration, the Committee on Publication Ethics guidelines (http://publicationethics.org/ accessed on 1 December 2024), and the REporting of studies Conducted using Observational Routinely-collected health Data (RECORD) Statement, validated by the Enhancing the Quality and Transparency of Health Research Network (www.equator-network.org/ accessed on 1 December 2024). No personal data that could lead to formal identification of the patient were stored in the databases. The study was not advertised. All patients diagnosed with EC, treated with primary surgery and underwent ProMisE, were enrolled in this prospective longitudinal observational study. Surgical staging, included total hysterectomy and bilateral salpingo-oophorectomy with lymph nodal staging, either using the sentinel lymphnode technique or systematic lymphadenectomy (in the pelvis and, when indicated, in the para aortic area), was performed for all patients. At our institution we offer sentinel lymph node biopsy in the first instance, plus backup lymphadenectomy in case of procedure failure, while we usually offer systematic lymphadenectomy in case of suspicious node at preoperative imaging or intraoperative examination and in case of intermediate–high preoperative risk factors. We excluded patients without adequate surgical staging, who received neoadjuvant chemotherapy or underwent surgery for recurrence EC. Baseline characteristics such as age, body mass index (BMI), parity, medical conditions, relevant comorbidities and surgical details were collected. Furthermore, histopathological details and data regarding adjuvant treatment were gathered. We investigated the need for adjuvant treatment according to classical histopathological risk factors (such as FIGO 2009 stage, histotype, grade, myometrial invasion and lymphovascular space invasion) and to National CCN guidelines 3.2024 [12] for endometrial malignancies (ENDO-4, ENDO-5 and ENDO-6), with a minimum level of evidence of category 2B (based upon lower-level evidence, there is NCCN consensus that the intervention is appropriate), and the age of the women. Afterward, we re-staged the included EC cases using the recent FIGO 2023 classification, evaluating any potential stage shifts. Based on the European Society for Medical Oncology (ESMO)/ESGO/European SocieTy for Radiotherapy & Oncology (ESTRO) risk classes, we hence investigated the need for adjuvant treatment according to the assigned risk class, assessing, in light of these changes, the modifications in adjuvant treatment decision-making [13].

All variables were compared among patients with FIGO 2009 I–II versus FIGO 2009 III–IV. Univariate analysis using Pearson, Chi-squared and Mann–Whitney U tests were conducted. Differences were considered statistically significant at a *p* value < 0.05. All statistical tests were performed using IBM SPSS for Windows, Version 23.

## 3. Results

During the study period, 211 women who underwent surgery for EC were enrolled. One-hundred and seventy-seven (84%) were finally staged as FIGO 2009 I–II and 34 (16%) as FIGO 2009 III–IV. The baseline characteristics of the women enrolled are represented in Table 1.

No statistical differences were noted comparing baseline characteristics in early versus advanced FIGO 2009 stages, in particular regarding BMI, menopausal status, the use of hormone replacement treatment (HRT) and the presence of abnormal uterine bleeding (AUB). Table 2 depicts the surgical outcomes of the whole population. For early and advanced FIGO 2009 stages, it can be seen that a minimally invasive approach was more frequent for early-stage disease (*p* < 0.01), as well as the use of the sentinel lymph node technique (*p* < 0.01). Conversely, para-aortic lymphadenectomy and omental biopsy were performed more frequently in the case of advanced stage.

The histopathological outcomes are represented in Table 3. The data show that in advanced stages, only 57.1% of patients have an endometrioid histotype compared with 84.7% of early-stage tumors; in addition, 60% of advanced tumors have G3 grading versus 23.9% of early-stages. Moreover, FIGO stage III–IV patients are more likely to have deep myometrial infiltration (85.6% vs. 42.1%), substantial lymph vascular space invasion (LVSI–71.4% vs. 31.8%), p53 abnormal expression at immunohistochemical stain (22.9% vs. 13.1%) and cervical involvement (40% vs. 5.7%). In contrast, the advanced stages are less likely to express estrogen and progesterone receptors (62.9% vs. 80.1% and 57.4% vs. 76.7%, respectively).

**Figure 1:**
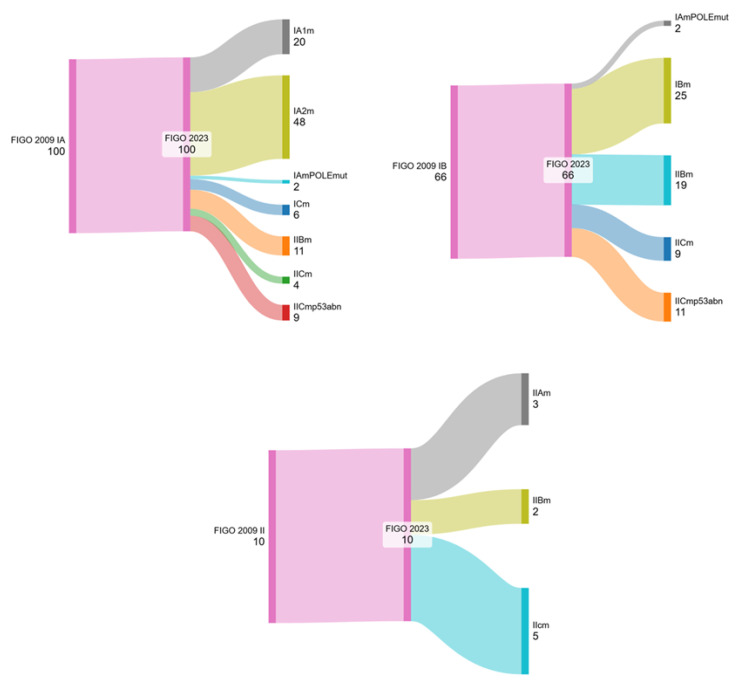
Sankey diagram shows how early-stage FIGO 2009 I–II patients are reallocated when staged according to the new FIGO staging 2023.

Adjuvant treatment was given to 86.7% of the study population: 107 women (50.7%) had vaginal brachytherapy (VBT), 30 women (14.2%) had external beam radiotherapy (EBRT) +/− VBT, none had chemotherapy alone and 46 women (21.8%) had sequential chemo-radiation treatment; specifically, all 35 patients in the advanced stages of the disease underwent chemo-radiotherapy treatment. Patients of our cohort were re-staged according to the new FIGO staging 2023; the population breakdown is reported in Table 4 and Figure 1.

As a matter of interest, we noted that the most relevant changes in the FIGO stages were observed in early endometrial cancer patients. As can be seen in Figure 1, the stage shift was more pronounced for FIGO 2009 IA and IB. In fact, based on Sankey’s diagrams, it can be seen that 20 (20%) patients remained at stage IA1 while 78 (78%) patients initially at stage IA according to FIGO 2009 had been upstaged and two patients (2%) were re-staged to FIGO 2023 IAmPOLEmut. In women initially staged as FIGO IB, we confirmed this stage in 25 (37.9%) patients, 2 patients (3%) were downstaged to FIGO 2023 IAmPOLEmut and the remaining 39 (59.1%) were all upstaged.

Using the FIGO 2009 staging system, there were ten patients classified as stage II. After re-staging with FIGO 2023, we observed a division among FIGO 2023 IIAm, IIBm and IICm, respectively, with three (30%), two (20%) and five (50%) patients (Figure 1).

Overall, in the early stages, considering the impact of the POLE and the P53 mutation, we found two patients were downstaged (1.13%) and 20 patients (11.4%) were upstaged; two patients previously staged as IA with FIGO 2009 were re-staged to IAmPOLEmut with FIGO 2023.

After the application of the FIGO 2023 classification, we re-evaluated the population and proposed a subdivision based on the ESMO/ESGO/ESTRO risk classes (Table 5) with the appropriate adjuvant treatment (Table 6).

According to Table 3 and Table 6, we investigated the changes in the need for adjuvant treatment with FIGO 2009 managed with NCCN guidelines 3.2024 compared to FIGO 2003 after the adoption of the ESMO/ESGO/ESTRO guidelines, specifically in the early-stages of endometrial cancer.

**Figure 2:**
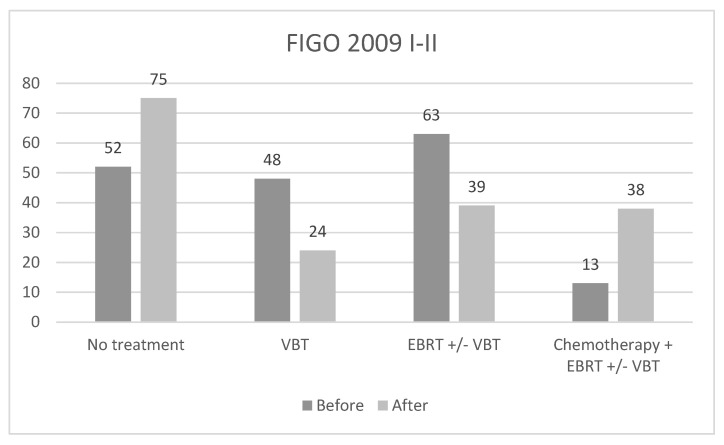
Adjuvant treatment in FIGO 2009 I–II, before and after re-staging according to FIGO 2023. FIGO: International Federation of Obstetrics and Gynecology; VBT: vaginal brachytherapy; EBRT: external beam radiotherapy. Values are expressed as *n*.

In FIGO 2009 I–II (Figure 2), 52 patients were eligible for no treatment and all of them were confirmed for follow-up.

In the VBT group, according to FIGO 2009, half of the patients changed to an adjuvant treatment indication. In fact, among 48 women candidates with exclusive VBT, 22 (45.8%) of them become eligible for exclusive follow-up, 5 (10.4%) for EBRT +/− VBT and 4 (8.3%) for Chemotherapy + EBRT +/− VBT; only 17 (35.4%) patients were confirmed eligible for VBT. According to FIGO 2009, 63 patients were eligible for EBRT +/− VBT (Figure 2) and after re-staging, 30 women changed to an adjuvant treatment indication. In detail, 22 (34.9%) become eligible for Chemotherapy + EBRT +/− VBT, 7 patients (11.1%) for VBT and 1 patient (1.6%) for exclusive follow-up. Of 13 women candidates for Chemotherapy + EBRT +/− VBT using FIGO 2009, 12 (92.3%) of them confirmed the indication and one (7.7%) was eligible for EBRT +/− VBT.

Overall, taking into account all the shifts in adjuvant treatment indications, in early-stage EC, there is a decreased relative risk (RR) to receive an adjuvant treatment using FIGO 2023 (RR 0.84; CI95% 0.74–0.95; *p* < 0.01) when compared to the FIGO 2009 classification. No significant risk (RR 1.01; CI95% 0.76–1.3; *p* = 0.08) for a more aggressive adjuvant treatment was noted after the FIGO 2023 re-staging.

In FIGO 2009 stage III–IV, 35 patients were eligible for Chemotherapy + EBRT +/− VBT, 9 of them were shifted towards chemotherapy and only 1 towards EBRT +/− VBT. No changes in the treatment modalities were observed for those women candidates who required chemotherapy according to FIGO 2009.

## 4. Discussion

Our study demonstrates the impact of the new FIGO 2023 classification on the categorization of various cases of endometrial cancer and on decision-making regarding adjuvant therapy. After applying ProMisE, as described by Vermij et al. [14], we found 5 patients with the POLEmut (2.4%), 33 patients with the p53 mutation (15.6%), 61 patients with MMRd (28.9%), and the remaining (112 patients—53.1%) fell into the NMSP class, representing the largest group in a percentage similar to what is described in the case studies of de Vitis et al. [15]. In Yu et al.’s case history, the molecular classification of a sample of 547 Chinese patients showed the presence of 69 POLEmut cases (12.6%), suggesting that molecular classes may also be influenced by ethnicity [16]. In our case study, most staging changes were noted among the most represented group of patients (Stages I–II), driven by the inclusion of molecular subtypes, the presence of LVSI, and the identification of more aggressive histological features. The same achievement was observed by Han et al. [17]. Also, in their case series, stage changes occurred among early-stage patients, and there were no cases observed where early-stage patients moved into the advanced stage group or vice versa. Regarding FIGO stage IA, in our case series, 31.4% of patients were upstaged due to the presence of LVSI, aggressive histotype and myometrial infiltration. This percentage is slightly higher than in the study by Öncü et al. in which upstaging was observed in 26.9%. This difference can be explained by the characteristics of the sample: in our case series, 9.8% of the 2009 FIGO stages had substantial LVSI vs. 2.6% in the case series of Öncü et al. Whereas in both our and Öncü’s case series, 13% of the 2009 FIGO IA stages had both an aggressive histotype and myometrial infiltration and were, therefore, upstaged to FIGO 2023 IIC [18]. Concerning the FIGO 2009 IB stage, in the work of Tsolakidis et al., 27/95 (28.4%) patients progressed from FIGO 2009 IB to FIGO 2023 IIC due to the presence of myometrial infiltration of an aggressive histotype; these results mirror what happened in our population in which 15/65 (23%) of the FIGO 2009 IB patients were upstaged to stage FIGO 2023 IIC [19]. In the study by Matsuo et al. [20], downstaging from FIGO 2009 stage IIIA to FIGO 2023 stage IA3 (observed in 22.2% of the cases) was reported. In our population, this did not occur, as no patient with ovarian endometrioid cancer exhibited the characteristics described in FIGO 2023 required for reclassification as IA3 [11,21]. Instead, these patients presented with ovarian extension or metastasis originating from the endometrial tumor.

Stage changes have led to a rethinking of the adjuvant therapy that can be proposed to patients. Stage IA1, IA2 patients and IB stage < 60-year-old patients have a low risk of recurrence and no adjuvant treatment is recommended. After applying the FIGO 2023 staging system, the number of stage II patients increases; according to the ESGO/ESTRO/ESTRO guidelines, stage II patients have high-intermediate risk of recurrence leading to the need for adjuvant EBRT +/− VBT or chemotherapy in the case of high grade histotypes. Due to the presence of substantial LVSI, 34 patients were upstaged from stage I FIGO 2009 to stage IIB FIGO 2023. One of the key aspects of the new FIGO 2023 classification is highlighting the negative impact of substantial LVSI on patients’ prognosis. In addition, the recent study of Dagher et al. [22,23] clearly shows that focal LVSI is associated with a worse 5 years PFS compared to no LVSI, calling for a revision of the FIGO staging. The increase in patients who are offered sequential chemo-radiotherapy treatment results from either the presence of the p53 mutation or the upstaging of stage I (2009) to stage II (2023), due to the presence of the endometrioid G3 histotype (even in the absence of the p53 mutation) [24]. The 2023 FIGO staging system has provoked extensive discussion within the scientific community, largely due to its perceived inadequacy in accurately reflecting disease extension. For instance, ovarian involvement in low-grade endometrioid carcinoma is classified as Stage IA3, despite being a poor prognostic factor often necessitating adjuvant therapy, even in low-risk scenarios [25]. Similarly, isolated tumor cells (ITC) in sentinel nodes are not associated with upstaging in endometrial cancer, although limited prospective data support observation-only approaches for ITC cases, underscoring the need for further research [25]. While molecular and genomic classifications offer significant potential for improved prognostication, their widespread clinical implementation is hindered by retrospective study designs and small sample sizes, which limit the strength of current evidence. As reported by Raspagliesi et al., the primary objective of a staging system is to enhance prognostic accuracy and guide the development of practical and effective treatment plans. However, the updated FIGO staging system appears to fall short in describing disease extent and presents challenges in clinical integration due to its complexity [25]. The inclusion of molecular analyses, such as POLE mutations and p53, has resulted in minor staging adjustments, with 11.4% of cases upstaged and approximately 1.13% downstaged. Notably, the molecular examination of seventeen women with EC was required to alter treatment recommendations for a single patient, emphasizing the importance of cost-effectiveness evaluations, particularly in resource-limited settings [23]. Furthermore, evidence suggests that molecular classification is especially critical for early-stage patients, as supported by Betella et al., while it offers limited therapeutic value for advanced-stage MMR-proficient patients [26]. Another controversial element concerns the way in which molecular classification is carried out: POLE sequencing and immunohistochemistry of p53 and MMR proteins is indicated as a surrogate for sequencing techniques (NGS) by the ProMisE study [14]. The aim was to make molecular classification as accessible as possible, although there is increasing evidence that the discrepancy between results of immunohistochemistry and NGS can range from 5% to 10% [27,28]. Despite this, even the immunohistochemistry analysis has not yet entered as a routine practice in all European hospitals [29] and the survey conducted by Zannoni et al. shows that less than 10% of Italian centers perform the NGS analysis of POLE [30]. The FIGO 2023 stage is, therefore, not applicable in most clinical centers and, moreover, is complicated and non-intuitive and this can introduce a few difficulties in clinical settings [31]. Surprisingly, the 2023 FIGO system does not attribute prognostic significance to the MMRd class, despite the availability of emerging therapeutic strategies for these tumors. Moreover, MSI status remains critical for therapeutic decision-making in both first-line advanced/metastatic disease and relapse settings. The latest trials conducted in this setting, in fact, have focused on the role of immunotherapy using MMR status as a discriminator of the population [32,33,34,35,36,37,38,39]. Finally, there is increasing evidence that the NSMP class may be stratified according to the expression of estrogen and progesterone receptors in a low or high risk category of recurrence; this detail is not considered in the current staging [40,41]. Other biomarkers, such as MUC16, ESR1, PGR, WFDC2, MKI67, ERBB2, L1CAM, CDH1, and PTEN, are under investigation to complement the prognostic capability of the ProMisE classification. However, none of these biomarkers have been validated or incorporated into current clinical guidelines, highlighting the need for further research and standardization [32]. It is noteworthy that FIGO deserves recognition for its extensive efforts in updating endometrial cancer staging, aligning it with the latest scientific advancements in molecular and genetic prognostic factors, even though with the arise of new challenges [42]. Based on this, every effort performed by the scientific community and stakeholders to validate its clinical usefulness are of utmost interest. While the sample size was substantial, the cohort’s restriction to a single center may have constrained the generalizability and validity of the results and hence it is recommended that further validation independent cohorts investigate the rate and modalities of adjuvant treatment after re-staging. In addition, the re-staging process and the choice of adjuvant therapy performed purely on the basis of disease characteristics does not consider comorbidities and general health status, which inevitably affect the choice of adjuvant therapy.

## 5. Conclusions

We demonstrate that implementing the FIGO 2023 staging system and molecular classification is crucial in early-stage EC due to significant shifts in treatment modalities. While the new staging system highlights many changes, it does not emphasize microsatellite status, making molecular classification essential. However, this approach does not account for the disparities in resource availability for its application. The greatest challenge remains integrating the new staging system with the existing adjuvant treatment guidelines, which are based on the previous staging classification. Therefore, we are looking forward to the updated ESMO and ESGO guidelines and how they will integrate the new staging and new evidence on target therapies, making the treatment proposal comprehensible and addressing all the prognostic factors of the disease.

## Figures and Tables

**Table 1:** Baseline characteristics of the population.

	All Patients (*n* = 211)	FIGO 2009 I–II (*n* = 176)	FIGO 2009 III–IV (*n* = 35)	*p*
Age	66 (59–75)	65 (58–76)	67 (59–74)	0.78
BMI	27.1 (23.15–32)	27.6 (23.3–32)	26 (21–31.6)	0.54
Diabetes	32 (15.2%)	27 (15.3%)	5 (14.3%)	0.93
Hypertension	119 (56.4%)	101 (57.4%)	18 (51.4%)	0.65
Nulliparity	41 (19.4%)	33 (18.8%)	8 (22.9%)	0.51
Cesarean section	24 (11.4%)	20 (11.4%)	4 (11.4%)	0.93
Menopause	179 (84.8%)	152 (86.4%)	27 (77.1%)	0.33
HRT	29 (13.7%)	25 (14.2%)	4 (11.4%)	0.71
AUB	145 (68.7%)	124 (70.5%)	21 (60%)	0.34
Previous pelvic surgery	52 (24.6%)	40 (22.7%)	12 (34.3%)	0.11
Previous pelvic radiotherapy	4 (1.9%)	3 (1.7%)	1 (2.9%)	0.62
Previous cervical surgery	5 (2.4%)	4 (2.3%)	1 (2.9%)	0.81

FIGO: International Federation of Obstetrics and Gynecology; BMI: body mass index; HRT: hormone replacement treatment; AUB: abnormal uterine bleeding. Values are expressed as median (interquartile range) or *n* (%).

**Table 2:** Surgical characteristics of the population.

	All Patients (*n* = 211)	FIGO 2009 I–II (*n* = 176)	FIGO 2009 III–IV (*n* = 35)	*p*
MIS	109 (51.7%)	104 (59.1%)	5 (14.3%)	<0.01
Duration of surgery	125 (42–315)	118 (42–257)	156 (61–315)	<0.01
SLN technique	82 (38.9%)	74 (41.8%)	5 (15.7%)	<0.01
BPLND	129 (61.1%)	106 (59.9%)	23 (67.6%)	0.4
PALND	27 (12.8%)	16 (9.1%)	11 (32.4%)	<0.01
Omental biopsy	33 (15.6%)	22 (12.4%)	11 (32.4%)	<0.01

FIGO: International Federation of Obstetrics and Gynecology; MIS: minimally invasive surgery; SLN: sentinel lymph node; BPLND: bilateral pelvic lymphadenectomy; PALND: para-aortic lymphadenectomy. Values are expressed as median (interquartile range) or *n* (%).

**Table 3:** The table summarizes histopathological and molecular features of all populations as well as the adjuvant treatment to which the patients underwent.

	All Patients (*n* = 211)	FIGO 2009 I–II (*n* = 176)	FIGO 2009 III–IV (*n* = 35)	*p*
Histotype				<0.01
Endometrioid	167 (79.1%)	146 (83.0%)	21 (60%)	
Serous	24 (13.6%)	17 (9.7%)	7 (20%)	
Clear-cell	6 (3.4%)	4 (2.3%)	2 (5.7%)	
Other	14 (7.9%)	8 (4.6%)	5 (11.4%)	
Grade				<0.01
G1	100 (47.4%)	97 (55.1%)	3 (8.6%)	
G2	47 (22.3%)	37 (21.0%)	10 (38.6%)	
G3	64 (30.3%)	42 (23.9%)	22 (62.9%)	
Myometrial invasion				<0.01
Absent/polyp/intramucosal	29 (13.7%)	28 (15.9%)	1 (2.9%)	
<50%	81 (38.4%)	75 (42.6%)	6 (17.1%)	
>50%	98 (46.4%)	73 (41.5%)	25 (71.4%)	
Serosal Breach	3 (1.4%)	0 (0%)	3 (8.6%)	
Cervical involvement	24 (11.4%)	10 (5.7%)	14 (40%)	<0.01
LVSI				<0.01
Negative	110 (52.1%)	101 (57.4%)	9 (25.7%)	
Focal	22 (10.4%)	20 (11.4%)	2 (5.7%)	
Substantial	79 (37.4%)	55 (31.3%)	24 (68.6%)	
Lymph nodal status				
Negative	190 (90%)	170 (96.6%)	20 (57.1%)	<0.01
ITC	7 (3.3%)	6 (3.4%)	1 (2.9%)	
Micrometastasis	2 (1%)	0 (0%)	2 (5.7%)	
Macrometastasis	12 (5.7%)	0 (0%)	12 (34.3%)	
PROmISE				
POLEmut	5 (2.4%)	4 (2.3%)	1 (2.9%)	
MMRd	61 (28.9%)	52 (29.5%)	9 (25.7%)	
p53abn	33 (15.6%)	25 (14.2%)	8 (22.9%)	
NSMP	112 (46.9%)	95 (54.0%)	17 (48.6%)	
IHC				
ER	164 (77.7%)	141 (80.1%)	23 (65.7%)	
PR	156 (73.9%)	135 (76.7%)	21 (60.0%)	
Adjuvant treatment (NCCN guidelines)				<0.01
No treatment	52 (24.6%)	52 (29.5%)	0 (0%)	
VBT	48 (22.7%)	48 (27.3%)	0 (0%)	
EBRT +/− VBT	63 (29.9%)	63 (35.8%)	0 (0%)	
Chemotherapy	6 (2.8%)	0 (0%)	6 (17.1%)	
Chemotherapy + EBRT +/− VBT	42 (19.9%)	13 (7.4%)	29 (82.9%)	

FIGO: International Federation of Obstetrics and Gynecology; NCCN: The National Comprehensive Cancer Network; LVSI: lymphovascular space invasion; ITC: isolated tumor cell; IHC: immunohistochemistry; ER: estrogen receptor; PR: progesterone receptor; VBT: vaginal brachytherapy; EBRT: external beam radiotherapy. Values are expressed as *n* (%).

**Table 4:** Re-staging according to FIGO 2023.

FIGO 2023	All Patients (*n* = 211)	FIGO 2009 I–II (*n* = 176)	FIGO 2009 III–IV (*n* = 35)
IA1	20	20 (11.4%)	0
IA2	48	48 (27.3%)	0
IA_mPOLEmut_	4	4 (2.3%)	0
IB	25	25 (14.2%)	0
IC	6	6 (3.4%)	0
IIA	3	3 (1.7%)	0
IIB	32	32 (18.2%)	0
IIC	18	18 (10.2%)	0
IIC_mp53abn_	20	20 (11.4%)	0
IIIA1	0	0	2 (5.7%)
IIIA2	5	0	5 (14.3%)
IIIB1	4	0	4 (11.4%)
IIIB2	2	0	2 (5.7%)
IIIC1i	1	0	1 (2.9%)
IIIC1ii	8	0	8 (22.9%)
IIIC2i	1	0	1 (2.9%)
IIIC2ii	3	0	3 (8.6%)
IVA	0	0	0
IVB	0	0	0
IVC	9	0	9 (25.7%)

FIGO: International Federation of Obstetrics and Gynecology; Values are expressed as *n* (%).

**Table 5:** Risk stratification according to FIGO 2023 stage with molecular status known and clinicopathological risk factors for early versus advanced FIGO 2009 stages.

	All Patients (*n* = 211)	FIGO 2009 I–II (*n* = 176)	FIGO 2009 III–IV (*n* = 35)
Low risk	68 (32.2)	68 (38.6%)	0
Intermediate risk	39 (18.5%)	39 (22.2%)	0
High-intermediate risk	49 (23.2%)	47 (26.7%	2 (5.7%)
High risk	46 (21.8%)	22 (12.5%)	24 (68.6%)
Advanced metastatic	9 (4.3%)	0	9 (25.7%)

FIGO: International Federation of Obstetrics and Gynecology. Values are expressed as *n* (%).

**Table 6:** Adjuvant treatment according to FIGO 2023 risk stratification with molecular status known.

	All Patients (*n* = 211)	FIGO 2009 I–II (*n* = 176)	FIGO 2009 III–IV (*n* = 35)
No treatment	75 (35.5%)	75 (42.6%)	0
VBT	24 (11.4%)	24 (13.6%)	0
EBRT +/− VBT	40 (19.0%)	39 (22.2%)	1 (2.9%)
Chemotherapy	9 (4.3%)	0	9 (25.7%)
Chemotherapy + EBRT +/− VBT	63 (29.9%)	38 (21.6%)	25 (71.4%)

FIGO: International Federation of Obstetrics and Gynecology; VBT: vaginal brachytherapy; EBRT: external beam radiotherapy. Values are expressed as *n* (%).

## Data Availability

The data presented in this study are available upon request from the corresponding author. The data are not publicly available due to privacy or ethical restrictions.
